# Tumor burden and management of basal cell carcinoma in Korean patients with Gorlin syndrome

**DOI:** 10.1016/j.jdin.2026.06.021

**Published:** 2026-07-20

**Authors:** Jinie Lee, Ji Su Lee, Soomin Park, Seong Jin Jo, Byung Jun Kim, Jee-Soo Lee, Moon-Woo Seong, Je-Ho Mun

**Affiliations:** a Seoul National University College of Medicine, Seoul, Republic of Korea; b Department of Dermatology, Seoul National University College of Medicine, Seoul, Republic of Korea; c Department of Dermatology, Seoul Metropolitan Government – Seoul National University (SMG-SNU) Boramae Medical Center, Seoul, Republic of Korea; d Department of Dermatology, Seoul National University Hospital, Seoul, Republic of Korea; e Institute of Human-Environment Interface Biology, Seoul National University, Seoul, Republic of Korea; f Department of Plastic Surgery, Seoul National University Hospital, Seoul National University College of Medicine, Seoul, Republic of Korea; g Department of Laboratory Medicine, Seoul National University Hospital, Seoul National University College of Medicine, Seoul, Republic of Korea; h Cancer Research Institute, Seoul National University, Seoul, Republic of Korea

**Keywords:** Asian population, basal cell carcinoma, epidemiology, Gorlin syndrome, hedgehog pathway inhibitor, Korea, nevoid basal cell carcinoma syndrome, PTCH1, tumor burden

*To the Editor:* Nevoid basal cell carcinoma syndrome (NBCCS), or Gorlin syndrome, is an autosomal dominant disorder characterized by multiple basal cell carcinomas (BCCs) and systemic manifestations.^1^ Registry-based studies in Western populations have demonstrated variability in BCC burden; however, data in Asian populations remain limited. We aimed to describe the clinical characteristics of Korean patients with NBCCS, focusing on BCC burden, age distribution, and treatment strategies.

We reviewed patients diagnosed with NBCCS at Seoul National University Hospital between 2001 and 2025. Diagnoses were confirmed by germline testing in probands or first-degree relatives. BCCs were included if histopathologically confirmed or clinically consistent with dermoscopic and photographic documentation. Data on demographics, tumor burden and distribution, genetic variants, and treatment modalities were collected. This study was approved by the Institutional Review Board of Seoul National University Hospital.

A total of 30 patients with NBCCS were included (Table I). The male-to-female ratio was 1:1.7, and the mean age at last follow-up was 36.9 years. Germline testing was performed in 28 patients, with *PTCH1* variants detected in 26 (92.9%). The remaining 2 patients were diagnosed based on clinical criteria and the presence of a germline *PTCH1* variant in a first-degree relative within the cohort.

**Table I:** Patient demographics, characteristics of BCC and genetic variants of NBCCS

Characteristics	Total (*N* = 30)
Demographics	
Sex, male:female (ratio)	11:19 (1:1.7)
Age (y) at last follow-up, mean ± SD	36.9 ± 21.0
Characteristics of BCCs	
Patients with BCCs (%)	23 (76.7)
Age (y) at first BCC, mean ± SD	30.6 ± 14.4
No. of BCCs, median (range)	14 (2-196)
Anatomical location of BCCs	
Head and neck	807
Trunk	27
Upper limb	23
Lower limb	11
Palm	5
Sole	0
No. of BCCs with histopathological confirmation (%)	691 (79.2)
Germline *PTCH1* variants	
Germline test done	28 (93.3)
Presence of any *PTCH1* variant (%, *N* = 28)	26 (92.9)
Variant types	
Nonsense variants	10
c.403C>T, p. Arg135∗ (pathogenic)	3
c.3223G>T, p. Gly1075∗ (likely pathogenic)	2
c.702C>A, p. Cys234∗ (likely pathogenic)	1
c.2422C>T, p. Gln808∗ (likely pathogenic)	1
c.2095C>T, p. Gln699∗ (pathogenic)	1
c.3081G>A, p. Trp1027∗ (likely pathogenic)	1
c.2802T>A, p. Tyr934∗ (pathogenic)	1
Splice-site variants	4
c.1347+1G>A (pathogenic)	2
c.3450-2A>G (likely pathogenic)	1
c.2251-3C>G (VUS)	1
Frameshift variants	5
c.2415dup, p. Val806Serfs∗23 (pathogenic)	4
c.290delA, p. Asn97Thrfs∗20 (pathogenic)	1
Large deletions	2
Exon 21-24 deletion (pathogenic)	1
Exon 2-25 deletion (pathogenic)	1
Missense variants	5
c.2479A>G, p. Ser827Gly (likely pathogenic)	1
c.3419T>G, p. Leu1140Arg (VUS)	2
c.1665T>A, p. Asn555Lys (VUS)	1
c.1847G>A, p. Ser616Asn (VUS)	1

*BCC*, Basal cell carcinoma; *NBCCS*, nevoid basal cell carcinoma syndrome; *VUS*, variant of uncertain significance.

BCCs developed in 76.7% of patients, with a mean age at first diagnosis of 30.6 years. A total of 873 BCCs were identified, with a median of 14 lesions per affected patient (range, 2-196), predominantly on the head and neck. Older age at last follow-up was associated with increased cumulative BCC count (incidence rate ratio, 1.022; 95% CI, 1.002-1.042; *P* = .034) (Fig 1). After adjusting for observation time, younger age at first BCC onset independently predicted higher tumor burden (incidence rate ratio, 0.956; 95% CI, 0.932-0.981; *P* = .001) (Supplementary Fig 1, available via Mendeley at https://data.mendeley.com/datasets/9h8hz7tkdn/2).

**Fig 1:**
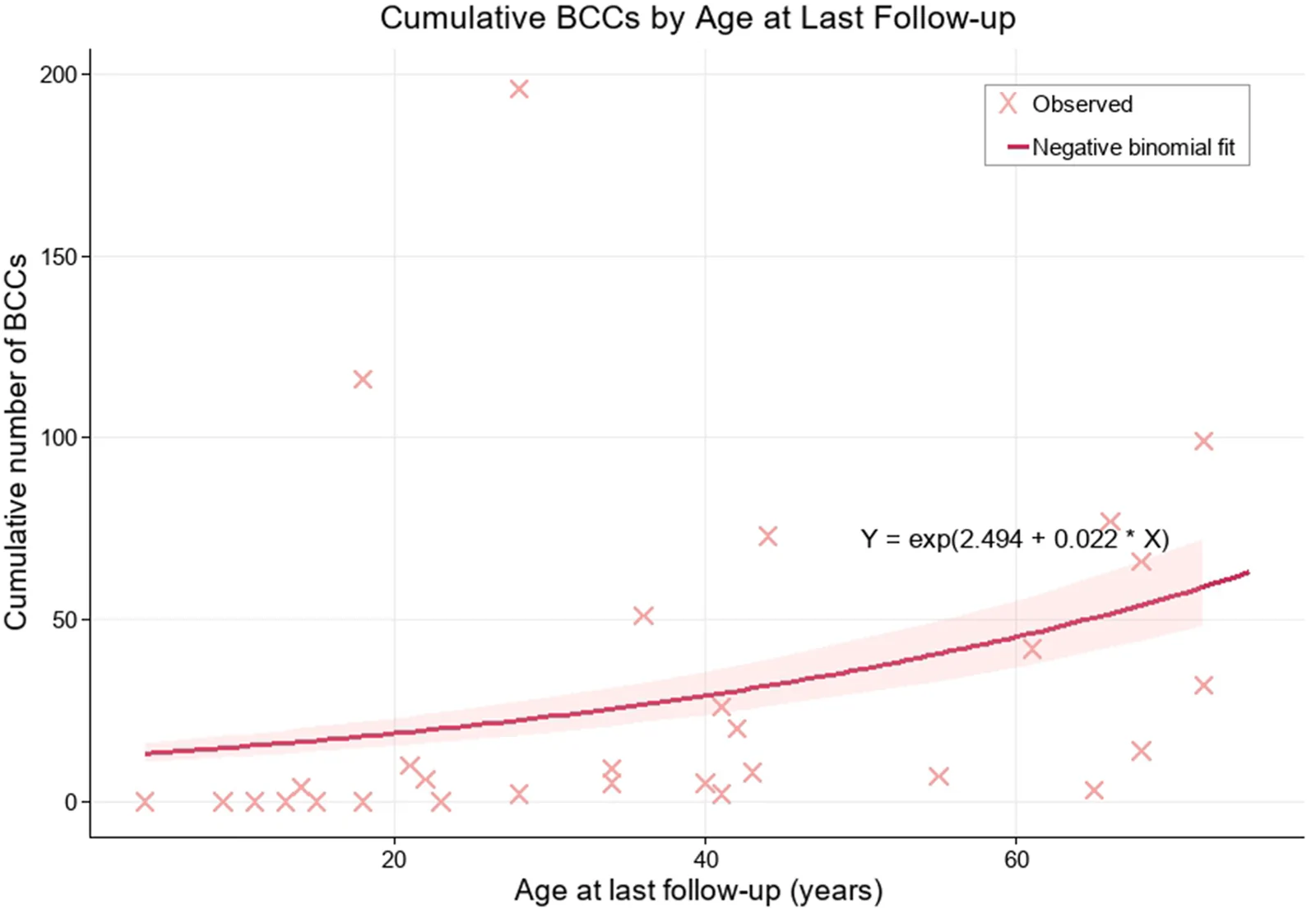
Cumulative basal cell carcinoma burden according to age at last follow-up in Korean patients with nevoid basal cell carcinoma syndrome. Each point represents an individual patient. The solid line represents the fitted negative binomial regression curve, and the shaded area indicates the 95% CI.

Compared with Western cohorts, Korean patients exhibited lower cumulative tumor burden and later onset. Prior US registry data reported a median of 160 BCCs at a mean age of 53 years,^2^ compared with 14 at 36.9 years in our cohort. When standardized to the same age, our model predicted a cumulative BCC count of 43.7 by age 53 years. These differences may reflect photoprotective effects of increased melanin, differences in UV exposure, and health care–related factors.^3^

Management remains challenging. All patients with BCCs underwent surgical excision, with adjunctive cryotherapy, CO_2_ laser, or photodynamic therapy used for multiple or recurrent lesions. Patients with high tumor burden required multimodal treatment; one refractory case received radiotherapy and oral itraconazole without significant improvement.^4^

Hedgehog pathway inhibitors have demonstrated high response rates in NBCCS-associated BCCs.^5^ However, hedgehog pathway inhibitors are not currently reimbursed by the Korean National Health Insurance System, limiting patient access. Expanding access to these agents in Asian countries could address a major unmet clinical need for patients with refractory NBCCS.

This study is limited by its retrospective single-center setting. Collectively, our findings delineate the clinical patterns and predictors of BCC burden in Korean patients with NBCCS and underscore the importance of early recognition, long-term surveillance, and access to targeted therapies for extensive or recalcitrant disease.

## Conflicts of interest

None disclosed.
